# One-qubit quantum gates in a circular graphene quantum dot: genetic algorithm approach

**DOI:** 10.1186/1556-276X-8-242

**Published:** 2013-05-16

**Authors:** Gibrán Amparán, Fernando Rojas, Antonio Pérez-Garrido

**Affiliations:** 1 Departamento de Física Aplicada, Antiguo Hospital de la Marina, Campo Muralla del Mar, UPCT, Cartagena, 30202, Murcia, Spain; 2 Departamento de Física Teórica, Centro de Nanociencias y Nanotecnologías, Universidad Nacional Autónoma de México, UNAM, Apdo, Postal 14, Ensenada, Baja California 22830, México

## Abstract

The aim of this work was to design and control, using genetic algorithm (GA) for parameter optimization, one-charge-qubit quantum logic gates *σ*_x_, *σ*_y_, and *σ*_z_, using two bound states as a qubit space, of circular graphene quantum dots in a homogeneous magnetic field. The method employed for the proposed gate implementation is through the quantum dynamic control of the qubit subspace with an oscillating electric field and an onsite (inside the quantum dot) gate voltage pulse with amplitude and time width modulation which introduce relative phases and transitions between states. Our results show that we can obtain values of fitness or gate fidelity close to 1, avoiding the leakage probability to higher states. The system evolution, for the gate operation, is presented with the dynamics of the probability density, as well as a visualization of the current of the pseudospin, characteristic of a graphene structure. Therefore, we conclude that is possible to use the states of the graphene quantum dot (selecting the dot size and magnetic field) to design and control the qubit subspace, with these two time-dependent interactions, to obtain the optimal parameters for a good gate fidelity using GA.

## Background

Quantum computing (QC) has played an important role as a modern research topic because the quantum mechanics phenomena (entanglement, superposition, projective measurement) can be used for different purposes such as data storage, communications and data processing, increasing security, and processing power.

The design of quantum logic gates (or quantum gates) is the basis for QC circuit model. There have been proposals and implementations of the qubit and quantum gates for several physical systems [1], where the qubit is represented as charge states using trapped ions, nuclear magnetic resonance (NMR) using the magnetic spin of ions, with light polarization as qubit or spin in solid-state nanostructures. Spin qubits in graphene nanoribbons have been also proposed. Some obstacles are present, in every implementation, related to the properties of the physical system like short coherence time in spin qubits and charge qubits or null interaction between photons, which is necessary to design two-qubit quantum logic gates. Most of the quantum algorithms have been implemented in NMR as Shor's algorithm [2] for the factorization of numbers. Any quantum algorithm can be done by the combination of one-qubit universal quantum logic gates like arbitrary rotations over Bloch sphere axes (*X*(*ϕ*), *Y*(*ϕ*), and *Z*(*ϕ*)) or the Pauli gates ($\sigma_{x}=\left(\begin{array}{cc} 0 & 1\\ 1 & 0 \end{array}\right),\sigma_{y}=\left(\begin{array}{cc} 0 & -i\\ i & 0 \end{array}\right),\sigma_{z}=\left(\begin{array}{cc} 1 & 0\\ 0 & -1 \end{array}\right)$) and two-qubit quantum gates like controlled NOT which is a genuine two-qubit quantum gate.

The implementation of gates using graphene to make quantum dots seems appropriate because this material is naturally low dimensional, and the isotope ^12^C (most common in nature) has no nuclear spin because the sum of spin particles in the nucleus is neutralized. This property can be helpful to increase time coherence as seen by the proposal of graphene nanoribbons (GPNs) [3] and Z-shape GPN for spin qubit [4].

In this work, we propose the implementation of three one-qubit quantum gates using the states of a circular graphene quantum dot (QD) to define the qubit. The control is made with pulse width modulation and coherent light which induce an oscillating electric field. The time-dependent Schrodinger equation is solved to describe the amplitude of being in a QD state *C*_*j*_(*t*). Two bound states are chosen to be the computational basis |0〉 ≡ |ψ_1/2_ |1〉 ≡ |ψ_− 1/2_ 〉 with *j* = 1/2 and *j* = −1/2, respectively, which form the qubit subspace. In this work, we studied the general n-state problem with all dipolar and onsite interactions included so that the objective is to optimize the control parameters of the time-dependent physical interaction in order to minimize the probability of leaking out of the qubit subspace and achieve the desired one-qubit gates successfully. The control parameters are obtained using a genetic algorithm which finds efficiently the optimal values for the gate implementation where the genes are: the magnitude (*ϵ*_0_) and direction (*ρ*) of electric field, magnitude of gate voltage (*V*_g0_), and pulse width (*τ*_v_). The fitness is defined as the gate fidelity at the measured time to obtain the best fitness, which means the best control parameters were found to produce the desired quantum gate. We present our findings and the evolution of the charge density and pseudospin current in the quantum dot under the gate effect.

## Methods

### Graphene circular quantum dot

The nanostructure we used consists of a graphene layer grown over a semiconductor material which introduces a constant mass term Δ [5]. This allows us to make a confinement (made with a circular electric potential of constant radio (*R*)) where a homogeneous magnetic field (*B*) is applied perpendicular to the graphene plane in order to break the degeneracy between Dirac's points *K* and *K*’, distinguished by the term *τ* = +1 and *τ* = −1, respectively.

**Figure 1:**
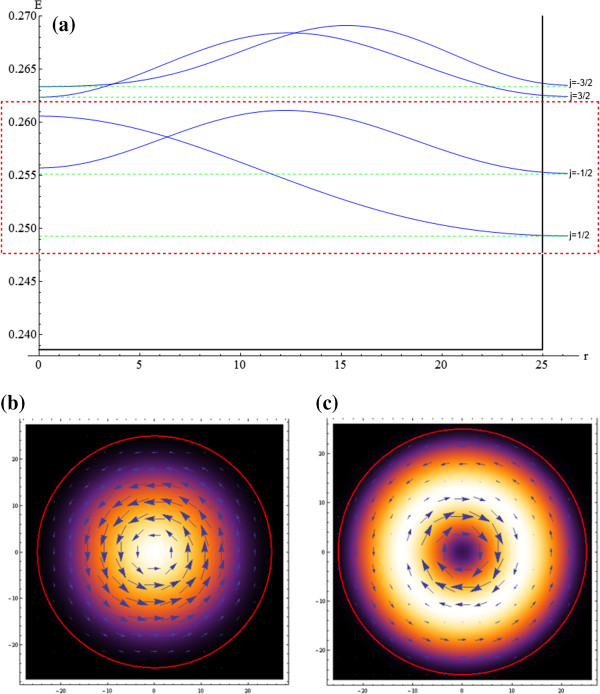
**Radial probability density (lowest states) and qubit subspace density and pseudospin current.** (**a**) Radial probability density plot for the four lowest energy states inside the graphene quantum dot with *R* = 25 nm and under a homogeneous magnetic field of magnitude *B* = 3.043 T. The selected computational basis (qubit subspace) is inside the red box. Qubit subspace spatial probability density plot and vector field of the pseudospin current in (**b**) |0〉 = |*ψ*_1/2_  and (**c**) |1〉 = |*ψ*_− 1/2_ , respectively.

The Dirac Hamiltonian with magnetic vector field in polar coordinates is given by [6]:

(1) $$\begin{array}{l} H_{0}\left(r,\varphi \right)=-\mathrm{iv}\sigma_{x}\left(\begin{array}{c} e^{\mathrm{i\varphi}}\\ e^{-\mathrm{i\varphi}} \end{array}\right)\frac{\partial }{\partial r}+v\sigma_{y}\left(\begin{array}{c} e^{\mathrm{i\varphi}}\\ e^{-\mathrm{i\varphi}} \end{array}\right)\left(\begin{array}{cc} \frac{\left(j-\frac{1}{2}\right)}{r}+\mathrm{br} & 0\\ 0 & \frac{\left(j+\frac{1}{2}\right)}{r}+\mathrm{br} \end{array}\right)\\ \;+\tau \Delta \sigma_{x}+U\left(r\right), \end{array}$$

where *v* is the Fermi velocity (10^6^ m/s), *b* = *eB*/2, and *j* which is a half-odd integer is the quantum number for total angular momentum operator *J*_z_. We need to solve $H_{\tau }\psi_{\left[j,\tau \right]}=E_{\left[j,\tau \right]}\psi_{\left[j,\tau \right]}$. Eigenfunctions have a pseudospinor form:

(2) $$\psi_{\left[j,\tau \right]}\left(r,\varphi \right)=e^{i\left(j-\frac{1}{2}\right)\varphi }\left(\begin{array}{c} \chi_{A}^{\tau }\left(r\right)\\ \chi_{B}^{\tau }\left(r\right)e^{\mathrm{i\varphi}} \end{array}\right),$$

where *χ* are hypergeometric functions *M* (a,b,z) and *U* (a,b,z) inside or outside of radius *R* (see [6] for details) (Figure 1).

Due to the constant mass term and broken degeneracy, we obtain two independent Hilbert spaces. Therefore, we can choose the space *K* for the definition of the computational basis of the qubit to implement the quantum gates and to make the dynamic control following a genetic algorithm procedure.

The wave function in graphene can be interpreted as a pseudospinor of the sublattice of atom type A or B. In order to visualize the physics evolution due to the gate operation, we calculate the pseudospin current as the expectation values for Pauli matrices $\bar{J}\left(r,\varphi ,t\right)=\left(j_{x},j_{y}\right)=v\psi^{*}\left(r,\varphi ,t\right)\bar{\sigma }\psi \left(r,\varphi ,t\right)$.

**Figure 2:**
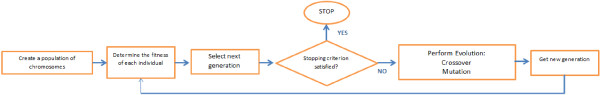
**Diagram of genetic algorithm.** Initial population of chromosomes randomly created; the fitness is determined for each chromosome; parents are selected according to their fitness and reproduced by pairs, and the product is mutated until the next generation is completed to perform the same process until stop criterion is satisfied.

The selected states that we choose to form the computational basis for the qubit are the energies (*E*_*j*_): *E*_1/2_ = .2492 eV and *E*_−1/2_ = .2551 eV (and the corresponding radial probability distributions is shown in Figure 2a). The energy gap is *E*_01_ = *E*_−1/2_ − *E*_1/2_ = 5.838 meV. To achieve transitions between these two states with coherent light, the wavelength required has to be $\lambda_{\mathrm{laser}}=\frac{2\mathrm{\pi c}\pi }{E_{01}}=212.35\;\mathrm{µm}$, which is in the range of far-infrared lasers. Also, in controlling the magnetic field *B*, it is possible to modify this energy gap. We present as a reference point the plot for the density probability and the pseudospin current for the two-dimensional computational basis |0〉 = |*ψ*_1/2_  (Figure 2b) and |1〉 = |*ψ*_− 1/2_  (Figure 2c), where a change of direction on pseudospin current and the creation of a hole (null probability near *r* = 0) is induced when one goes from qubit 0 to1.

#### Quantum control: time-dependent potentials

First of all, we have to calculate the matrix representation of the time-dependent interactions in the QD basis. Then, we have to use the interaction picture to obtain the ordinary differential equation (ODE) for the time-dependent coefficient which is the probability of being in a state of the QD at time *t* and finally obtaining the optimal parameter for gate operation.

##### Electric field: oscillating

These transitions can be induced by a laser directed to the QD carrying a wavelength that resonates with the qubit states in order to trigger and control transitions in the qubit subspace. We introduce an electric dipole interaction [7] using a time periodic Hamiltonian with frequency *ω*: *V*_laser_(*t*) = *e****ϵ*(*t*)***r*, with parameters ***ϵ*(*t*)** = ***ϵ***_**0**_ cos *ωt*, ***ϵ***_**0**_ = *ϵ*_0_(cos *ρ*, sin *ρ*), and *r* = *r*(cos *φ*, sin *φ*), the term *ρ is* the direction and *ϵ*_0_ is the magnitude of the electric field and are parameters constant in time. To determine the matrix of dipolar transitions on the basis of the QD states, the following overlap integrals must be calculated:

(3) $$V_{\mathrm{laser}}{}_{\mathrm{lj}}\left(t\right)=\overset{2\pi }{\underset{0}{\int }}\overset{\infty }{\underset{0}{\int }}\psi_{l}^{*}\left(r,\varphi \right)\;\left(\epsilon \left(t\right)\cdot r\right)\;\psi_{j}\left(r,\varphi \right)r\mathrm{dr}\mathrm{d\varphi},$$

where *l* and *j* are the state indices. In Equation 3, the radial part defines the magnitude of the matrix component, the angular part defines transition rules, and as a result, we get a non-diagonal matrix; this indicates that transitions are only permitted between neighbor states. The matrix components are complex numbers; ***ϵ***_0_ directed in $\hat{y}$ direction is a pure imaginary number and directed in $\hat{x}$ is a real number.

##### Voltage pulse on site

This interaction can be applied as a gate voltage inside the QD. In order to modify the electrostatic potential, we use a square pulse of width *τ*_v_ and magnitude *V*_g0_. The Hamiltonian is

(4) $$V_{\mathrm{gate}}\left(t\right)=V_{g0}\theta \left(-t+\tau_{v}+t0\right)\theta \left(t-t0\right)\theta \left(R-r\right),$$

(5) $$V_{\mathrm{gate}}{}_{\mathrm{lj}}=V_{g0}\delta_{l,j}\overset{R}{\underset{0}{\int }}\chi_{l}^{*}\left(r\right)\chi_{j}\left(r\right)\;r\mathrm{dr}.$$

The matrix components in Equation 5 are diagonal, so this interaction only modifies the energies on the site. Since the Heaviside function *θ* depends on *r* in Equation 4, the matrix components are the probability to be inside the quantum dot which is different for each eigenstate, so this difference can introduce relative phases inside the qubit subspace.

#### One-qubit quantum logic gates

Therefore, we have to solve the dynamics of QD problem in N-dimensional states involved, where the control has to minimize the probability of leaking to states out of the qubit subspace in order to approximate the dynamic to the ideal state to implement correctly the one-qubit gates. The total Hamiltonian for both quantum dot and time-dependent interactions is $H\left(t\right)=H_{0}+V_{\mathrm{gate}}\left(t\right)+V_{\mathrm{laser}}\left(t\right)$, where $H_{0}$ is the quantum dot part (Equation 1) and *V*_laser_(*t*) and *V*_gate_(*t*) are the time control interactions given by Equations 3 and 4.

We expand the time-dependent solution in terms of the QD states (Equation 2) $\psi \left(r,\varphi ,t\right)=\underset{l}{\sum }C_{l}\left(t\right)\psi_{l}\left(r,\varphi \right)$ as. Therefore, the equations for the evolution of probability of being in state l at time *t*, *C*_*l*_(*t*), in the interaction picture, are given by:

(6) $$\begin{array}{l} i\frac{\partial }{\partial t}C_{l}\left(t\right)=V_{g0}V_{\mathrm{gate}}{}_{l}\;\theta \left(-t+\tau_{v}+t0\right)\theta \left(t-t0\right)C_{l}\left(t\right)\\ \;+\epsilon_{0}\cos\left(\omega_{c}t\right)\underset{j}{\sum }\mathrm{Vl}V_{\mathrm{laser}}{}_{\mathrm{li},j}C_{j}\left(t\right)e^{i\left(E_{l}-E_{j}\right)t}. \end{array}$$

The control problem of how to produce the gates becomes a dynamic optimization one, where we have to find the combination of the interaction parameters that produces the one-qubit gates (Pauli matrices). We solve it using a genetic algorithm [8] which allows us to avoid local maxima and converges in a short time over a multidimensional space (four control parameters in our case). The steps in the GA approach are presented in Figure 2, where the key elements that we require to define four our problem are chromosomes and fitness.

In our model, the chromosomes in GA are the array of values {*V*_g0,_*τ*_v,_***ϵ***_**0**,_*ρ*}, where *V*_g0_ is the voltage pulse magnitude, *τ*_v_ is the voltage pulse width, *ϵ*_0_ is the electric field magnitude, and *ρ* is the electric field direction. The fitness function, as a measure of the gate fidelity, is a real number from 0 to 1 that we define as fitness(*t*_med_) = | < *Ψ*_obj_|*Ψ*(*t*_med_) > |^2^ × | < Ψ_0_|Ψ(2*t*_med_) > |^2^ where |Ψ_obj_ 〉 is the objective or ideal vector state, which is product of the gate operation (Pauli matrix) on the initial state |*Ψ*_0_〉. Then, we evolve the dynamics to the measurement time *t*_med_ to obtain |*Ψ*(*t*_med_)〉. Determination of gate fidelity results in the probability to be in the objective vector state at *t*_med_. Fitness involves gate fidelity at *t*_med_ and probability to be in the initial state at 2 *t*_med_. This gives a number between 0 and 1, indicating how effective is the transformations in taking an initial state to the objective state and back to the initial state in twice of time (the reset phase).

The initial population of chromosomes ({*V*_g0,_*τ*_v,_*ϵ*_0,_*ρ*}) is randomly created, then fitness is determined for each chromosome (which implies to have the time-dependent evolution of *C*_*l*_(*t*) to the measurement time); parents are selected according to their fitness and reproduced by pairs, and the product is mutated until the next generation is completed; one performs the same process until a stop criterion is satisfied.

## Results and discussion

**Figure 3:**
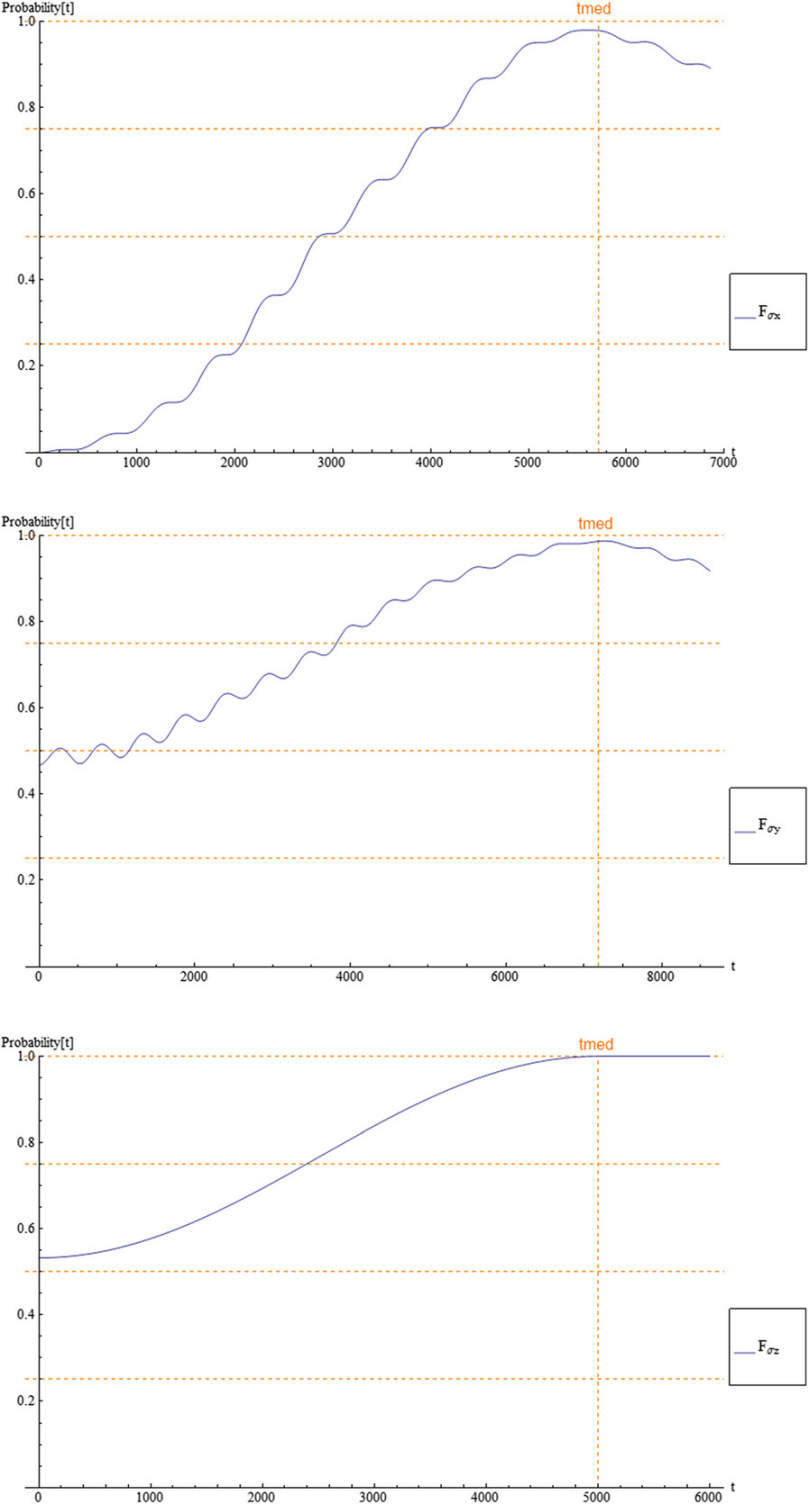
**Time evolution of gate fidelity or fitness for the three gates.** Plot of gate fidelity *σ*_x_ in the top side, *σ*_y_ in the middle, and *σ*_z_ in the bottom side; gate fidelity (*F*_*σ*I_ in blue where I is{x,y,z}) is the probability to be in the objective vector state; measurement time is shown in orange.

**Figure 4:**
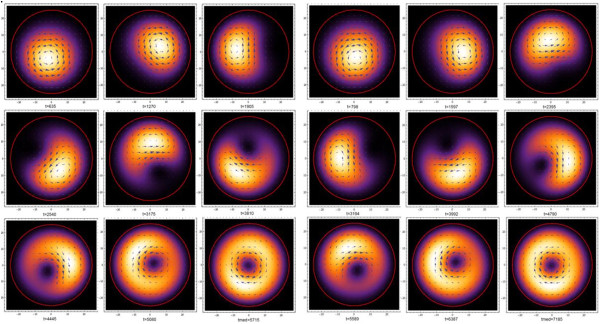
**Time evolution of probability density and pseudospin current for the quantum gate *σ***_**x **_**and *σ***_**y **_**operation.** Time evolution of density and current probability due to the effect of the produced quantum gate *σ*_x_ in the left side and *σ*_y_ in the right side, initial state |*Ψ*_0_〉 = |0〉 (Figure 1b).

The control dynamics were done considering *N* = 6 states, two of them are used as the qubit basis, so that the effect of the interaction stays inside the qubit subspace . The gate operation is completed in a time window that depends on *ϵ*_*0*_, and control parameters are defined to achieve operation inside a determined time window. The possible values of the electric field direction *ρ* is set from 0 to 2*π*, pulse width *τ*_v_ domain is set from 0 to time window and the magnitude *V*_g0_ is set from 0 to an arbitrary value. The genetic algorithm procedure is executed for quantum gates *σ*_x_ and *σ*_y_. The fitness reaches a value close to 1 near to 30 generations for both gates. The optimal parameters found for quantum gate *σ*_x_ are *V*_g0_ *=* .0003685, *τ*_v_ = 4215.95, *ϵ*_0_ = .0000924, and *ρ* = .9931*π*. For *σ*_y_ are *V*_g0_ = .0355961, *τ*_v_ = 326.926, *ϵ*_0_ = .0000735, and *ρ* = 1.5120*π*. For the quantum gate *σ*_z_, genetic algorithm is not needed because for this case, *ϵ*_*0*_ = 0, so Equation 6 is an uncoupled ordinary differential equation (ODE) with specific solution. To achieve this gate transformation in a determined time window, we can calculate *V*_g0_, so that the control values for this quantum gate are *V*_g0_ *=* .1859, *τ*_v_ = 5,000, *ϵ*_0_ = 0, and *ρ* = 0*.* In Figure 3, we plot the time evolution of the gate fidelity or fitness for the three gates. We observe a good optimal convergence close to 1 at the time of measurement and reaching again the reset phase. To see the state transition and the quantum gate effect in the space, it is convenient to plot the density probability in the quantum dot and the corresponding pseudospin current, where we see how the wave packet has different time trajectory according to the gate transformation. For instance, the direction and time of creation of the characteristic hole (null probability) in the middle of the qubit one, which correspond more or less to an equal superposition of the qubit zero and one (column 2 and row 2 in Figure 4, right). This process has to be different for *σ*_y_ because it introduces an imaginary phase in the evolution which is similar with the change of the arrow directions in the pseudospin current. The same situation arises for *σ*_z_ (result not shown), but in this case, we use as an initial state $\left|\Psi_{0}\right)〉=\frac{1}{\sqrt{2}}\left(\left|0,〉\right)+\left|1,〉\right)\right)$, which is similar to the plot of column 2 and row 2 in Figure 4 (left) and then to show explicitly the gate effect of introducing the minus in the one state to reach a rotated state similar to plot of column 2 and row 2 in Figure 4 (right).

## Conclusions

We show that with a proper selection of time-dependent interactions, one is able to control or induce that leakage probability out of the qubit subspace in a graphene QD to be small. We have been able to optimize the control parameters (electric field and gate voltage) with a GA in order to keep the electron inside the qubit subspace and produce successfully the three one-qubit gates. In our results, we appreciate that with the genetic algorithm, one can achieve good fidelity and found that little voltage pulses are required for *σ*_x_ and *σ*_y_ and improve gate fidelity, therefore making our proposal of the graphene QD model for quantum gate implementation viable. Finally, in terms of physical process, the visualization of the effects of quantum gates *σ*_x_ and *σ*_y_ is very useful, and clearly, both achieve the ideal states. The difference between them (Figure 4) is appreciated in the different trajectories made by the wave packet and pseudospin current during evolution due to the introduction of relative phase made by gate *σ*_y_.

## Competing interests

The authors declare that they have no competing interests.

## Authors’ contributions

The work presented here was carried out collaboration among all authors. FR and APG defined the research problem. GA carried out the calculations under FR and APG's supervision. All of them discussed the results and wrote the manuscript. All authors read and approved the final manuscript.
